# Bronchiolitis Obliterans and Primary Ciliary Dyskinesia: What Is the Link?

**DOI:** 10.7759/cureus.15591

**Published:** 2021-06-11

**Authors:** Ninoshka M Caballero-Colón, Yuhong Guan, Haiming Yang, Shuying Zhao, Wilfredo De Jesús-Rojas

**Affiliations:** 1 Department of Pediatrics, San Juan Bautista School of Medicine, Caguas, PRI; 2 Department of Respiratory Medicine, Beijing Children’s Hospital, Capital Medical University, National Center for Children’s Health, Beijing, CHN; 3 Department of Pediatrics, Medical Sciences Campus, School of Medicine, University of Puerto Rico, San Juan, PRI; 4 Department of Pediatrics, Ponce Health Sciences University, School of Medicine, Ponce, PRI

**Keywords:** bronchiolitis obliterans, primary ciliary dyskinesia, pseudomonas aeruginosa, dnah1, pediatrics

## Abstract

Bronchiolitis obliterans (BO) is a rare form of chronic obstructive lung disease characterized by obliteration of the small airways caused by inflammation and fibrosis. In children, BO most commonly appears following a severe lower respiratory tract infection. This phenomenon has been described as post-infectious BO (PIBO). PIBO presents with dyspnea, tachypnea, and persistent hypoxemia, as well as characteristic radiographic findings on high-resolution CT (HRCT) of the lungs. A few *DNAH1 *genetic variants have been postulated to have a role in the development of BO in patients with primary ciliary dyskinesia (PCD), but there is limited evidence regarding this, and etiologies are uncertain. PCD is a genetically heterogeneous autosomal recessive disorder characterized by ciliary dysfunction that causes impaired mucociliary clearance, leading to bronchiectasis and recurrent lower respiratory tract infections due to several pathogenic organisms including *Pseudomonas aeruginosa*. The link between rare PCD genetic variants and BO remains undetermined. We report the first case in Puerto Rico with Pseudomonal PIBO as the initial presentation of PCD; the patient was a four-year-old male. We also engage in a comparison of our case with previously reported cases of PIBO in PCD patients.

## Introduction

Bronchiolitis obliterans (BO) is a rare chronic obstructive lung disease that leads to permanent and irreversible obstruction of the small airways following chronic inflammation [1]. BO is characterized by partial or complete occlusion of the terminal respiratory bronchioles caused by inflammation and irreversible fibrosis [2]. Severe lower respiratory tract infection appears to be the most common etiology of BO in children and has been described as a separate clinical entity known as post-infectious BO (PIBO) [2]. PIBO is seen after infections caused by respiratory viruses such as influenza, parainfluenza, respiratory syncytial virus (RSV), metapneumovirus, and most commonly adenovirus serotypes 3, 7, and 21 [1-3]. Less common etiologies include infections due to *Mycoplasma pneumoniae*, *Legionella pneumophila*, *Bordetella pertussis*, Measles virus, human immunodeficiency virus-1 (HIV-1), and *Cytomegalovirus* [1]. Additionally, *Pseudomonas aeruginosa* infection has been associated with BO development in post-transplant settings [4]. Clinical presentation of BO is characterized by tachypnea, hypoxemia, productive cough, fixed polyphonic wheezes on auscultation, and characteristic radiographic imaging findings of mosaic pattern on high-resolution CT (HRCT) of the lungs. Diagnosis can be established by a combination of patient history and physical examination findings along with radiographic imaging [1,2]. A possible rare association between PIBO and primary ciliary dyskinesia (PCD) has been described in certain genetic PCD variants like *DNAH1 *[5]. However, the relationship between specific PCD genetic variants and a predisposition to developing BO in patients with PCD is still not established.

PCD is a genetic disorder of motile cilia with autosomal recessive inheritance, with more than 50 genes associated with the condition [6,7]. This disease is associated with neonatal respiratory distress, recurrent and chronic infections of the upper and lower respiratory tract, persistent wet cough, bronchiectasis, infertility, and laterality defects in 50% of cases [6]. Diagnosis of PCD is challenging due to its genetic variability, clinical presentation, and a lack of accessibility to diagnosis tools [7]. *Pseudomonas aeruginosa* is a common opportunistic pathogen in patients with PCD [8]. It has been associated with worse clinical outcomes in patients with bronchiectasis, a common lung complication in PCD patients [9]. However, it remains unclear as to what extent colonization with *Pseudomonas aeruginosa* affects the lung structure and function in patients with PCD in the long term [8]. To date, only seven patients with PIBO as the initial presentation of PCD have been described in the medical literature [5], and only three of them had *DNAH1* genetic variants. In this report, we describe an atypical presentation of PCD with a single heterozygous pathogenic *DNAH1 *genetic variant in a pediatric Puerto Rican patient with mosaic attenuation pattern and mild bronchiectasis on HRCT of the lungs. The patient findings were consistent with PIBO secondary to *Pseudomonas aeruginosa* chronic pulmonary infection.

## Case presentation

**Figure 1:**
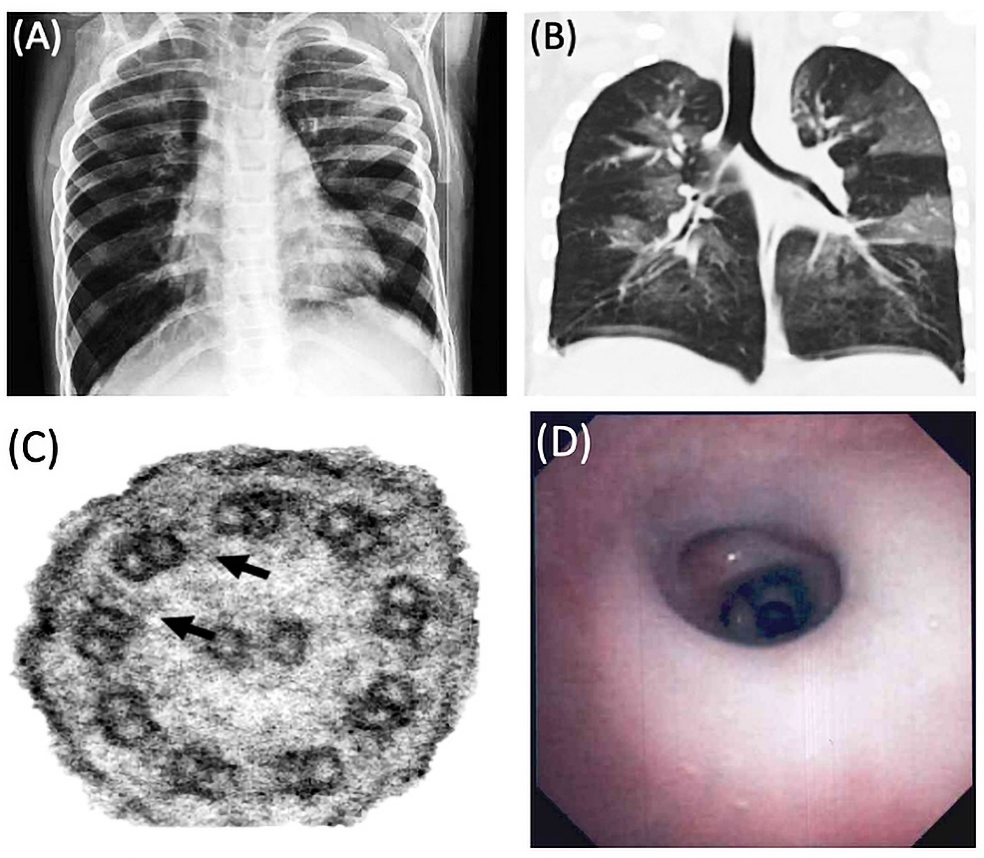
Chest X-ray, HRCT of the lungs, EM findings of nasal ciliary epithelium, and bronchoscopy evaluation of the patient (A) Hyperexpanded lungs with diffuse peribronchial thickening and bibasilar atelectasis. (B) Coronal view of HRCT of the lungs showing a mosaic attenuation pattern, bibasilar mild bronchiectasis, and air trapping. (C) Absence of inner dynein arm (arrows). Outer dynein arm was seen only on 20-25% of cross-sections in our patient. EM findings were consistent with PCD mutations in DNAI1 c.370C>T (p.Arg124Cys) and DNAH1 c.1610G>A (p.Ser537Asn). (D) Bronchoscopic image at the bronchus intermedius with purulent and thick secretions HRCT: high-resolution computed tomography; EM: electron microscopy; PCD: primary ciliary dyskinesia

The patient was a four-year-old male who presented with year-round wet cough, fatigue, and dyspnea on exertion for one year. His past medical history was remarkable for eight hospitalizations and six admissions to the pediatric intensive care unit (PICU) due to recurrent episodes of severe lower respiratory tract infections. The patient had been born at term with no history of neonatal respiratory distress syndrome, congenital heart defects, or major complications at birth. There was no history of recurrent sinus or ear infections during early infancy. Upon physical examination, vital signs were remarkable for pulse oximeter saturation at 88% on room air. Chest wall inspection revealed increased work of breathing and moderate intercostal and subcostal retractions. Lung auscultation revealed bibasilar crackles and expiratory polyphonic wheezes bilaterally. No cyanosis or clubbing was noted on extremities. Due to clinical instability and signs of acute respiratory failure, the patient was admitted to the PICU for acute management. Chest radiograph showed hyperexpanded lungs with diffuse bilateral peribronchial thickening and bibasilar atelectasis (Figure 1A). HRCT of the lungs revealed a mosaic attenuation pattern, bibasilar mild bronchiectasis, and air trapping concerning for BO (Figure 1B). The patient underwent a diagnostic flexible fiberoptic bronchoscopy with bronchoalveolar lavage (BAL), which revealed purulent and thick secretions causing mucus plugs at distal airways (Figure 1D). Analyzed BAL was positive for *Pseudomonas aeruginosa* resistant to first-generation cephalosporins. The BAL cytology demonstrated suppurative polymorphonuclear infiltrate with degenerative changes consistent with acute bacterial pneumonia. Differential cell count showed the following findings - neutrophils: 94%, macrophages: 4%, lymphocytes: 1%, and eosinophils: 1%. Based on clinical findings, the patient was managed with double antipseudomonal coverage for two weeks, followed by inhaled tobramycin for 28 days as an attempt toward *Pseudomonas aeruginosa* eradication. Pulmonary function tests (PFTs) showed the following results - forced vital capacity (FVC): 39%, forced expiratory volume (FEV1): 38%, FEV1/FVC: 96%, and forced expiratory flow (FEF) 25-75%: 27% of percentage predicted, consistent with a restrictive airflow pattern at baseline.

Based on patient history, physical examination, and imaging findings of bronchiectasis and positive chronic *Pseudomonas aeruginosa* infection, genetic testing for cystic fibrosis and PCD was performed. Results from an extended genetic panel, which evaluated deletions and duplication on 35 PCD-related genes plus mutations on the cystic fibrosis transmembrane conductance regulator (CFTR) gene, identified a single heterozygous pathogenic variant in *DNAI1* c.370C>T (p.Arg124Cys) and a single heterozygous variant of uncertain significance (VUS) in *DNAH1 *c.1610G>A (p.Ser537Asn). A nasal ciliary biopsy, at baseline, was evaluated by an independent pathologist who noticed a lack of inner dynein arms and partial absence of outer dynein arms in 20-25% of cross-sections, which were concordant with genetic findings (Figure 1C). All presented findings suggested the diagnosis of PCD in our patient as per the PCD diagnostic guidelines published by the American Thoracic Society and the PCD Foundation [10]. Sweat test and mutations on the CFTR gene were negative. Basic immunologic evaluation and genetic panel for immunodeficiencies were not contributory. Abdominal sonogram and barium swallow to evaluate for laterality defects and gastroesophageal reflux disease respectively were negative.

## Discussion

PCD is characterized by the abnormal ciliary function associated with mutations in more than 200 structural proteins, leading to defective beating patterns and dysfunctional mucociliary clearance [6]. The *DNAH1* gene encodes an axonemal inner dynein arm heavy chain. Mutations in this gene have been associated with autosomal recessive multiple morphological abnormalities of the sperm flagella, a form of asthenozoospermia that leads to male infertility [11,12]. Imtiaz et al. have reported a novel biallelic homozygous variant of *DNAH1* c.3460 A>C (p.Lys1154Gln) in two sisters with PCD who were born from a consanguineous union [13]. They found that molecular variation in *DNAH1* may play a role in PCD [13]. The *DNAH1* c.1610G>A (p.Ser537Asn) variant in our patient resulted in a missense mutation that replaced serine with asparagine at the codon 537 of the DNAH1 protein. This variant causes small physiochemical differences between the two amino acids but has not been described in the medical literature as a cause of *DNAH1 *gene-related disease as of today. The other variant in our patient, *DNAI1 *c.370C>T (p.Arg124Cys), replaces arginine with cysteine at the codon 124 of the DNAI1 protein. While the outcome of this mutation is uncertain, this variant has been described as pathogenic in the medical literature. The clinical significance of the VUS *DNAH1* c.1610G>A (p.Ser537Asn) and the pathogenic *DNAI1* c.370C>T (p.Arg124Cys) genetic variant reported in our patient are inconclusive, which makes the diagnosis of PCD difficult in challenging cases from populations with high genetic variability, such as Puerto Ricans. Previous studies have suggested that the severity of pulmonary disease in patients with PCD is associated with specific genes and the interaction of different mutations in the same gene [14,15]. However, in our case, mutations were found in different genes. Altogether, this prompted us to question whether the *DNAH1* genetic variant could increase the susceptibility to develop BO in patients with PCD.

**Figure 2:**
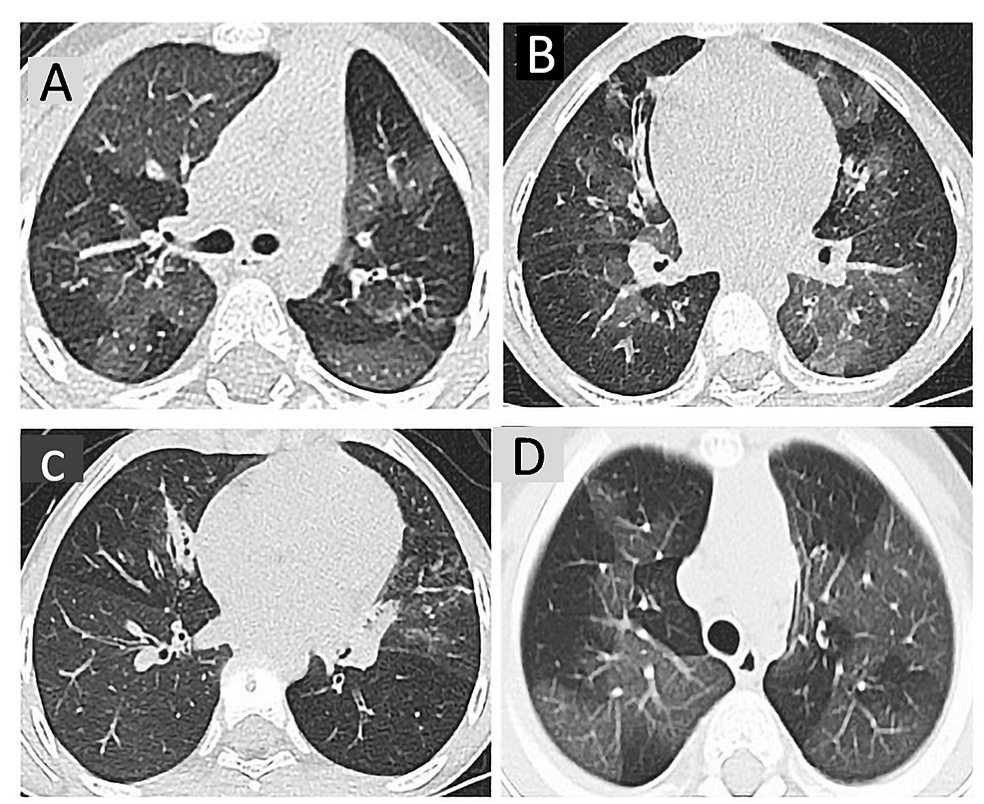
HRCT images from cases presented in Table 1 HRCT images A, B, and C correspond to bronchiolitis obliterans and primary ciliary dyskinesia cases 1-3 in Table 1 (previously reported cases in the medical literature). HRCT image D corresponds to case 4 in Table 1 (case presented in this manuscript) Images 2B and 2C were reprinted with permission from Guan et al. [5] HRCT: high-resolution computed tomography

Given the genetic heterogeneity in Puerto Rico and clinical variability of PCD, this rare condition is often underdiagnosed, leading to further progression of lung damage in affected patients [6,7]. Previous studies have reported that 70% of patients had seen a medical provider more than 50 times before a diagnosis of PCD was confirmed [16]. This fact highlights the importance of a better understanding of the spectrum of clinical manifestations of PCD in children. Recently, Guan et al. described seven pediatric patients from China with PIBO as an initial presentation of PCD [5]. In this study, patients were found to have findings of mosaic attenuation patterns, bronchiectasis, atelectasis, and air trapping on HRCT of the lungs. They showed moderate to severe obstructive airflow on PFTs. Interestingly, a higher incidence of PIBO was found in patients with *DNAH1 *variant alleles [5]. These findings are comparable with our case. Genetic and clinical characteristics of previously published cases [5] are presented in Table 1 and Figure 2.

In Guan et al.'s study, the infectious etiology of PIBO on *DNAH1 *was adenovirus and mycoplasma. In our case, findings related to BO were concomitantly found with a BAL positive for *Pseudomonas aeruginosa* pulmonary infection. A study by Vos et al. has reported that patients with *Pseudomonas* infection are more likely to develop BO in post-transplant settings [4]. For instance, PCD genetic testing for mutation in *DNAH1* should be considered in patients with findings of mosaic attenuation pattern suggestive of PIBO in HRCT of the lungs if clinically indicated. Furthermore, bronchoscopy with BAL may be considered in these patients to exclude concomitant infectious processes like those related to *Pseudomonas aeruginosa* as an etiology.

Additional research is required to better understand the role of *Pseudomonas aeruginosa* in PIBO pathogenesis and the link with PCD secondary to *DNAH1 *genetic mutations. This also raises the question as to whether genetic predisposition and microbiome may interact, increasing the risk of PIBO in PCD patients, and this relates to the goal of identifying patients at risk of worse long-term pulmonary complications to prevent further lung damage using an individualized treatment approach. The study of the genotypic and phenotypic characterization of patients with *DNAH1 *may help to finally confirm the association of specific mutations like* DNAH1 *with the development of PIBO in patients with PCD.

**Table 1:** Cases of bronchiolitis obliterans and primary ciliary dyskinesia patients with mutations in the DNAH1 gene Cases 1-3: bronchiolitis obliterans and primary ciliary dyskinesia cases previously reported in the medical literature. Case 4: the case presented in this study HRCT: high-resolution computed tomography; PCD: primary ciliary dyskinesia

Case	Clinical manifestations and physical findings	Affected PCD gene	Base change	Amino-acid change	Zygosity	HRCT
1	15-month-old male presenting with wheezing after moderate physical activity two months after adenovirus pneumonia	DNAH1	c.5356C>T c.1286+7C>A	p.R1786C P?	Compound Heterozygous	Diffuse lobar areas of lung attenuation (Figure 2A)
2	Four-year-old male with recurrent episodes of wheezing and exercise intolerance two months after pneumonia of unknown etiology	DNAH1	c.2912G>A c.11135G>A	p.R971H p.R3712	Compound Heterozygous	Bilateral pulmonary infiltrates and bronchiectasis (Figure 2B)
3	Seven-year-old female with year-round wet cough, recurrent episodes of pneumonia, and exercise intolerance	DNAH1	c.2610G>A	p.W870X	Homozygous	Lobar regions of mosaic attenuation with bilateral infiltrates (Figure 2C)
4	Four-year-old male with year-round wet cough, dyspnea, persistent hypoxemia, wheezing, and crackles	DNAI1 DNAH1	c.370C>T c.1610G>A	p.Arg124Cys p.Ser537Asn	Heterozygous	Bilateral mosaic attenuation pattern, and air trapping (Figure 2D)

## Conclusions

This case report highlights the importance of including PCD in the differential diagnosis of patients presenting with *Pseudomonas aeruginosa* infection and BO. Radiographic findings, including mosaic attenuation pattern on HRCT of the lungs in a patient with *Pseudomonas aeruginosa* infection, should raise a high index of suspicion for both PIBO and PCD. Awareness of the variable clinical manifestations in populations with heterogenous genetics and initial presentations of PCD will contribute immensely to early diagnosis and appropriate medical management to prevent or delay further lung disease. Additional research to understand the link between *Pseudomonas aeruginosa* and *DNAH1* genetic variants on the development of PIBO is required. A better understanding of the specific PCD genetic variants associated with PIBO may help to create new evidence-based diagnostic strategies and the development of individualized medical treatments to improve the quality of life of patients with rare pulmonary diseases like PCD.
